# Effect of a DACC-coated dressing on keratinocytes and fibroblasts in wound healing using an in vitro scratch model

**DOI:** 10.1007/s10856-022-06648-5

**Published:** 2022-02-08

**Authors:** Bianka Morgner, Johanna Husmark, Anna Arvidsson, Cornelia Wiegand

**Affiliations:** 1 Department of Dermatology, University Hospital Jena, Jena, Germany; 2 ABIGO Medical AB, Askim, Sweden

## Abstract

Wound dressings that exert an antimicrobial effect in order to prevent and treat wound infections can be harmful to the wound healing process. Dressings with hydrophobic coatings, however, have been suggested to both reduce the microbial load and promote the healing process. Therefore, the potential effects of a dialkylcarbamoyl chloride (DACC)-coated dressing on fibroblasts and keratinocytes in wound healing were studied using mechanical scratch wounding of confluent cell layers as an in vitro model. Additionally, gene expression analysis by qRT-PCR was used to elucidate the longitudinal effects of the DACC-coated dressing on cell responses, specifically inflammation, growth factor induction and collagen synthesis. DACC promoted cell viability, did not stick to the cell layers, and supported normal wound healing progression in vitro. In contrast, cells became attached to the uncoated reference material, which inhibited scratch closure. Moreover, DACC slightly induced *KGF*, *VEGF*, and *GM-CSF* expression in HaCaT cells and NHDF. Physiological *COL1A1* and *COL3A1* gene expression by NHDF was observed under DACC treatment with no observable effect on *S100A7* and *RNASE7* levels in HaCaT cells. Overall, the DACC coating was found to be safe and may positively influence the wound healing outcome.

**Figure:**
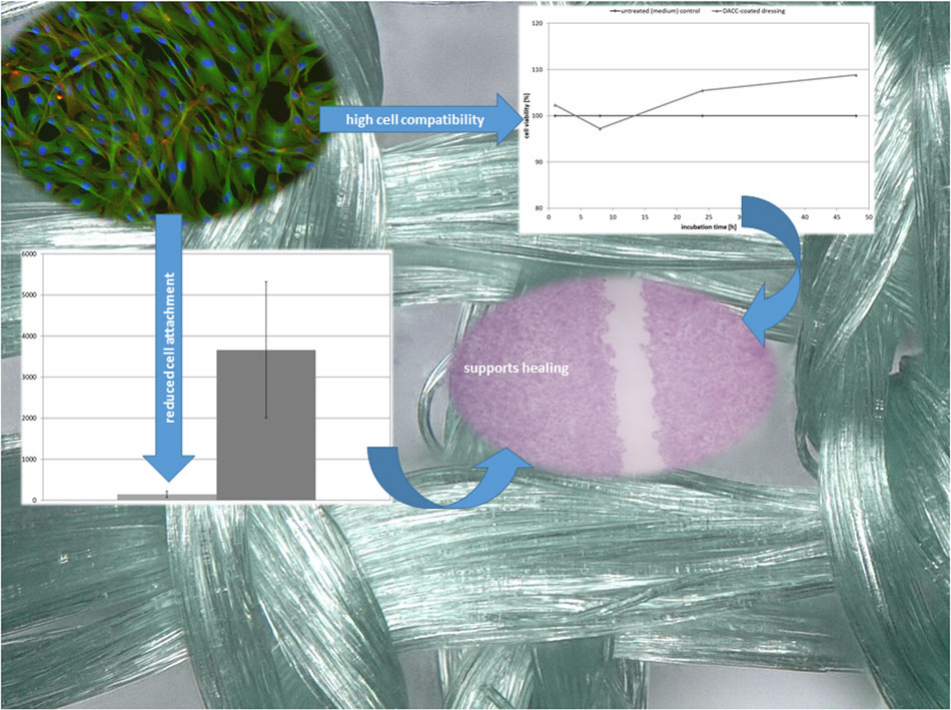
Graphical abstract

Graphical abstract

## Introduction

The increasing antimicrobial resistance seen worldwide [1] demands novel concepts on how to prevent wound infection. Physical binding of microorganisms by wound dressings as a “passive” antimicrobial method may have advantages over killing bacteria by an “active” mechanism, since the latter leads to bacterial cell wall disruption and may release bacterial endotoxins that increase the inflammatory response in nonhealing wounds [2, 3]. Hydrophobization of surfaces by, for example coating of fibers with dialkylcarbamoyl chloride (DACC), a fatty acid derivative, can convey a high binding capacity for bacteria [4], which use high cell surface hydrophobicity (CSH) for adhesion to tissue surfaces [5]. Hydrophobic antimicrobial dressings have been suggested for the treatment and prevention of wound infections as well as for wound bioburden reduction [6, 7]. At the same time it is vital to avoid any negative effects on the wound healing process. Wound healing is a complex, highly regulated process entailing diverse epidermal and dermal cells among white blood cells involved in inflammatory reactions, which are controlled by various cytokines and growth factors [8]. Any dressing that is applied to a wound makes close contact with the cells engaged in wound healing processes. Thus, it is important to elucidate potential interactions between these cells and wound dressings. A prominent feature of these dressings are hemostatic effects, such as inducing or accelerating the coagulation cascade locally [9], without causing adverse events that include foreign body reactions, infection, and granuloma formation [10]. Moreover, dressing materials could fuse to the wound surface due to drying drainage, the ingrowth of new tissue or a sticky surface, which causes pain and tissue trauma upon removal and impedes healing [11]. These features are most commonly related to fibroblasts and keratinocytes. Fibroblasts produce extracellular matrix components and fill the wound with newly synthesized tissue [12]. After tissue injury, they migrate to the injury site and begin depositing collagen [13]. Epidermal keratinocytes can act as non-professional immune cells and secrete a wide range of pro-inflammatory cytokines and chemokines, including IL-1α, IL-6, CXCL8 (IL-8), TNF-α, and TGF-β, to recruit neutrophils and promote a T-cell derived immune response [14, 15]. They are also important effectors in restoring the barrier function of the skin. Cytotoxic properties that may affect cell proliferation and cell migration, and thus delay wound healing, are especially important to note [13, 16, 17]. Conversely, the positive effects of dressings on cellular reactions can be analyzed using in vitro methods monitoring cell morphology [18] and cell layer regeneration [19]. Non-adhering dressings have been shown to exert a positive influence due to their non-adherent surface features that allow atraumatic removal and their ability to stimulate cells in the wound environment [20]. Furthermore, material combinations of cotton or polyester with vaseline and several silicone dressings are well known for their ability to avoid damaging newly synthesized tissue during dressing changes and effectively stimulating wound healing [21]. Recently, Falk and Ivarsson demonstrated that a DACC-coated dressing potentially increases fibroblast proliferation and migration in vitro and it has been proposed that such dressings may further promote the healing process in hard-to-heal wounds [22].

This study is the first to explore the potential effects of the DACC-coated dressing on fibroblasts as well as its influence on the human keratinocyte cell line HaCaT employing the mechanical scratch wounding of confluent monolayers as a model to study wound healing in vitro. Scratch wound models allow the direct measurement of cell migration and cell layer regeneration comparable to healing processes that occur at the wound margins [19]. These models have recently been shown to be adaptable, such that they allow investigating the effects of wound dressings [21]. It is also the first study to comprehensively investigate the safety of applying DACC-coated dressings and to determine any potential damaging or cytotoxic effects on keratinocytes and fibroblasts that are involved in wound healing by measuring their cellular metabolic activity, any reduction of which indicates cytotoxic effects [16, 17]. Furthermore, gene expression analysis by qRT-PCR enabled the specific longitudinal effects of the DACC-coated dressing on wound healing in vitro to be determined. Employing several in vitro methods may help to explain current clinical observations. Here, accomplished comprehensive investigational methodology based on an array of different in vitro tests was used to demonstrate, for the first time, the effects of the DACC-coated dressing on normal human dermal fibroblasts (NHDF) and human keratinocytes (HaCaT cells) associated with promoting cell layer regeneration and wound healing processes in vitro.

## Materials and methods

### Materials

Green-colored, cellulose acetate fabric was either treated in an impregnation step to become DACC-coated (Sorbact^®^ compress) or kept uncoated and used as reference material (RM). Both, the DACC-coated and uncoated reference material (provided by the manufacturer Abigo Medical AB, Askim, Sweden) were sterilized by gamma sterilization. Several material aspects are identical for the Sorbact^®^ compress and the reference fabric, i.e., both fabrics consist of a sparse mesh allowing fluid absorption into secondary layers and conform easily to the wound bed. However, after DACC-coating, the fabric features a high hydrophobicity and low absorbing properties, similar to the polypropylene stress control. In contrast, the reference fabric is hydrophilic and absorbs water to some extent.

### Cultivation of keratinocytes and fibroblasts

The HaCaT cells (provided by Prof. Fusenig, Heidelberg) and normal human dermal fibroblasts (NHDF; Promocell) were cultured in Dulbecco′s modified Eagle’s Medium (DMEM; AMIMED) supplemented with 1% antibiotic-antimycotic solution (AMIMED) and 10% fetal calf serum (FCS; PAN). Cells were cultured for 7 days in cell culture flasks (75-cm^2^, Greiner bio-one, Germany) at 37 °C and in a humidified atmosphere containing 5% CO_2_ [21].

### Sample preparation

Wound dressing samples were cut aseptically corresponding to 1.5 cm × 1.5 cm and were used directly for testing [21]. Sterilized polypropylene pieces (Nunc™ Cap Mats, ThermoScientific) of the same size were used as a stress control to simulate the mechanical strain by application of the dressing material,.

### Determination of the effect on cell viability

Cell viability after contact with either the DACC-coated dressing or the uncoated reference was determined [21]. In brief, cells were harvested through trypsin-EDTA (Gibco) treatment, seeded into 4-well culture slides (BD Biosciences) at a density of 40,000 cells/cm^2^, and cultured for 48 h to confluence. Stress controls and material samples were placed directly onto the cell layers. DMEM alone served as untreated (medium) controls. Cells were incubated for 1, 8, 24, and 48 h. Subsequently, the number of viable, active cells was determined using the photometric MTT assay (MTT Cell Proliferation Assay Kit, Invitrogen) according to the manufacturer’s recommendations. The absorbance was measured at 580 nm using a microplate photometer (POLARstar Galaxy, BMG Labtech). The number of viable cells was calculated as percentage of the medium control.

### Assessment of fibroblast adherence to the material samples

Cell adherence to material surface of samples removed from the NHDF monolayer after 1, 24, and 48 h from the previous experiment (see “Determination of the effect on cell viability”) was also investigated. Cell presence was ascertained using a luminometric adenosine triphosphate (ATP) assay (ATPLite^TM^-M Assay, PerkinElmer) that determines the cellular ATP concentration based on light generated during the ATP-dependent conversion of luciferin by luciferase. Luminescence was measured using a microplate luminometer (LUMIstar Galaxy, BMG Labtech, Germany). The ATP concentrations were calculated based on a standard curve and the number of viable cells specified as cellular ATP-content in [nM].

### Scratch wound assay

Scratch wound closure under the DACC-coated dressing and the uncoated RM samples was determined [21]. Briefly, HaCaT cells and fibroblasts were harvested through trypsin-EDTA treatment, seeded into 4-well culture slides at a density of 40,000 cells/cm^2^, and cultured for 48 h to confluence before the layers were scratched with a sterile pipette tip. Stress controls and material samples were placed directly onto the scratch while DMEM alone served as the untreated (medium) control. Cell scratches were incubated for 1, 8, 24, and 48 h. Material samples and stress controls were then removed and cells were stained with hematoxylin and eosin for evaluation. Microscopic assessment was done using the VHX 950F digital microscope (KEYENCE DEUTSCHLAND GmbH) and images were obtained. Scratch width was determined using the VHX 950F software (KEYENCE DEUTSCHLAND GmbH).

### Gene expression analysis

Gene expression analysis of specific inflammatory cytokines, growth factors, antimicrobial peptides, and collagen was performed at 4, 8, 24, and 48 h [23]. In brief, human cells were lysed by adding RLT buffer (Qiagen) containing 10 µl/ml β-mercaptoethanol after removal of the cell culture supernatants and subsequently incubated for 3 min on ice and 3 min under shaking. Lysates were loaded onto QIA Shredder spin columns (Qiagen), and centrifuged at 4 °C and 10,000 × *g* for 2 min. Subsequently, automatic RNA purification was performed using the RNeasy^®^ Mini Kit and the QIAcube (Qiagen). RNA concentration was determined at 260 nm using the SPECTROstar^®^ Omega with an UV/Vis plate (BMG Labtech GmbH). For reverse transcription the High Capacity cDNA Reverse Transcription Kit by Applied Biosystems (ThermoScientific™) was used according to the manufacturers’ instructions. The PCR protocol was run using the Mastercycler^®^ gradient thermal cycler (Eppendorf), which included primer annealing for 10 min at 25 °C, reverse transcription for 120 min at 37 °C, and termination for 5 min at 85 °C. The cDNA samples were stored at −80 °C until further use. After reverse transcription, cDNA was diluted to obtain a final test concentration of 0.5 ng/ml. Gene expression was analyzed by RTqPCR using the QuantiNova™ SYBR Green PCR Kit (Qiagen) following the manufacturers’ instructions. Briefly, master mix prepared on ice contained forward and reverse primer (each test concentration 0.5 µM) and cDNA or Yellow Template Dilution Buffer as a no template control. Using the qTOWER3G (Analytik Jena AG) the real-time amplification protocol was set for polymerase heat activation at 95 °C for 3 min, and 40 cycles with three steps: denaturation at 95 °C for 5 s, annealing at 57 °C for 10 s, and elongation at 72 °C for 10 s. Signals were detected at λex/λem 470 nm/520 nm. Finally, a melting curve from 65 °C to 95 °C served as amplicon control. All primer sequences or ordering IDs are shown in Table 1.

**Table 1:** The primer sequences and ordering IDs used for the SYBR Green-based RTqPCR

Target gene	Ordering IDs/primer sequence		Manufacturer
*ACTB*	Hs_ACTB_1_SG QuantiTect^®^ Primer Assay		Qiagen GmbH
*CXCL8*	Hs_CXCL8_1_SG QuantiTect^®^ Primer Assay		
*TGFB1*	Hs_TGFB1_1_SG QuantiTect^®^ Primer Assay		
*FGF2*	Hs_FGF2_1_SG QuantiTect^®^ Primer Assay		
*PDGFA*	Hs_PDGFA_1_SG QuantiTect^®^ Primer Assay		
*PDGFC*	Hs_PDGFC_1_SG QuantiTect^®^ Primer Assay		
*RNASE7*	Hs_RNASE7_1_SG QuantiTect^®^ Primer Assay		
*COL1A1*	Hs_COL1A1_1_SG QuantiTect^®^ Primer Assay		
*COL1A3*	Hs_COL1A3_1_SG QuantiTect^®^ Primer Assay		
*IL1A*	Fw	5′-CGCCAATGACTCAGAGGAAGA-3′	Eurofins Genomics
	Rev	5′-AGGGCGTCATTCAGGATGAA-3′	
*IL6*	Fw	5′-CCACCGGGAACGAAAGAGAA-3′	
	Rev	5′-GAGAAGGCAACTGGACCGAA-3′	
*CXCL1*	Fw	5′-TCACCCCAAGAACATCCAAAG-3′	
	Rev	5′-GAGTGTGGCTATGACTTCGGTTT-3′	
*GM-CSF*	Fw	5′-TGAACCTGAGTAGAGACACTGC-3′	
	Rev	5′-GCTCCTGGAGGTCAAACATTTC-3′	
*VEGF*	Fw	5′-AAAACACAGACTCGCGTTGC-3′	
	Rev	5′-GGCTTGTCACATCTGCAAGTAC-3′	
*EGF*	Fw	5′-AAGTTGTACTGGTGCGATGC-3′	
	Rev	5′-TTCGGCGTTTTGAACCATCC-3′	
*S100A7*	Fw	5′-GTCCAAACACACACATCTCACT-3′	
	Rev	5′-TCATCATCGTCAGCAGGCTT-3′	

### Statistical analysis

Experiments were performed in triplicate. Measurements were carried out in quadruplicate (scratch assay) or duplicate (MTT, ATP, gene expression) and all values expressed as the mean ± SD (standard deviation). Statistical significance was determined using one-way analysis of variance (ANOVA, Microsoft Excel 2000). A differences was considered statistically significant at *p* < 0.05. Asterisks indicate significant deviations at the respective time point from the untreated control (*p < 0.05; **p < 0.01; ***p < 0.001) unless otherwise stated.

## Results

### Influence of DACC and uncoated reference material on cell viability

**Fig. 1:**
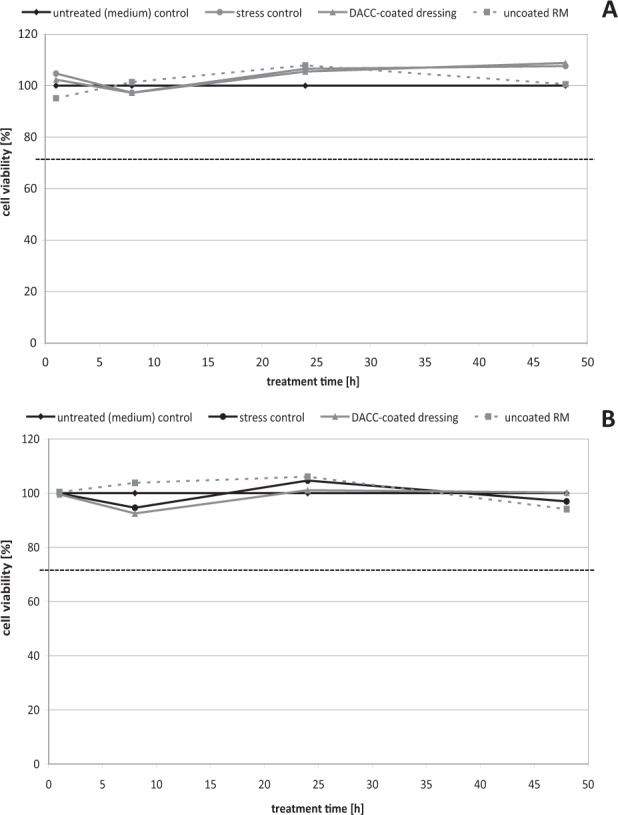
Determination of the influence of the stress control, DACC-coated dressing, and uncoated RM on HaCaT cell viability (**A**) and NHDF viability (**B**) compared to the untreated (medium) control using the MTT test

**Fig. 2:**
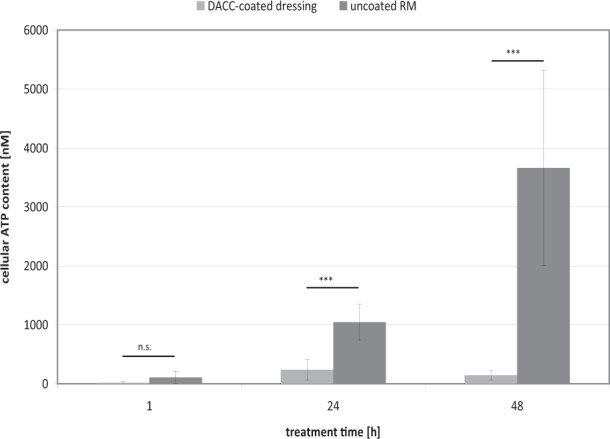
Comparison of NHDF adherence to the DACC-coated dressing samples and uncoated RM determined by measurement of the cellular ATP-content using the luminometric ATP assay. Asterisks indicate a significant deviation between DACC-coated dressing and uncoated RM (*p < 0.05; **p < 0.01; ***p < 0.001), while n.s. designates “not significant”

Viability of HaCaT cells (Fig. 1A) and NHDF (Fig. 1B) after contact with DACC and uncoated RM over 48 h was evaluated using the photometric MTT test. The stress control exhibited no negative influence on cell viability in the assays. The DACC-coated dressing exhibited high cell compatibility in vitro. There was no significant effect of DACC on the viability of HaCaT cells (Fig. 1A) and NHDF (Fig. 1B) demonstrating a cell viability of ~100% similar to the untreated control. Uncoated RM had no effect on the metabolic activity of HaCaT cells and NHDF, thus, also demonstrating a cell viability of ~100% similar to the untreated control. No significant differences between the DACC-coated dressing, uncoated RM and the stress control compared to the untreated control were observed. However, cells became attached to the uncoated fibers of the RM in contrast to the DACC-coated dressing. Cell attachment to the surfaces of the dressings after their removal from the cell layers was determined by ATP measurement (Fig. 2). The ATP levels found on DACC were very low, while those on uncoated RM were significantly higher reaching five-times the concentration found on DACC-coated dressings after 24 h (*p* < 0.001) and almost 25 times the concentration after 48 h (*p* < 0.001).

### Effect of DACC on normal wound healing progression in vitro

**Fig. 3:**
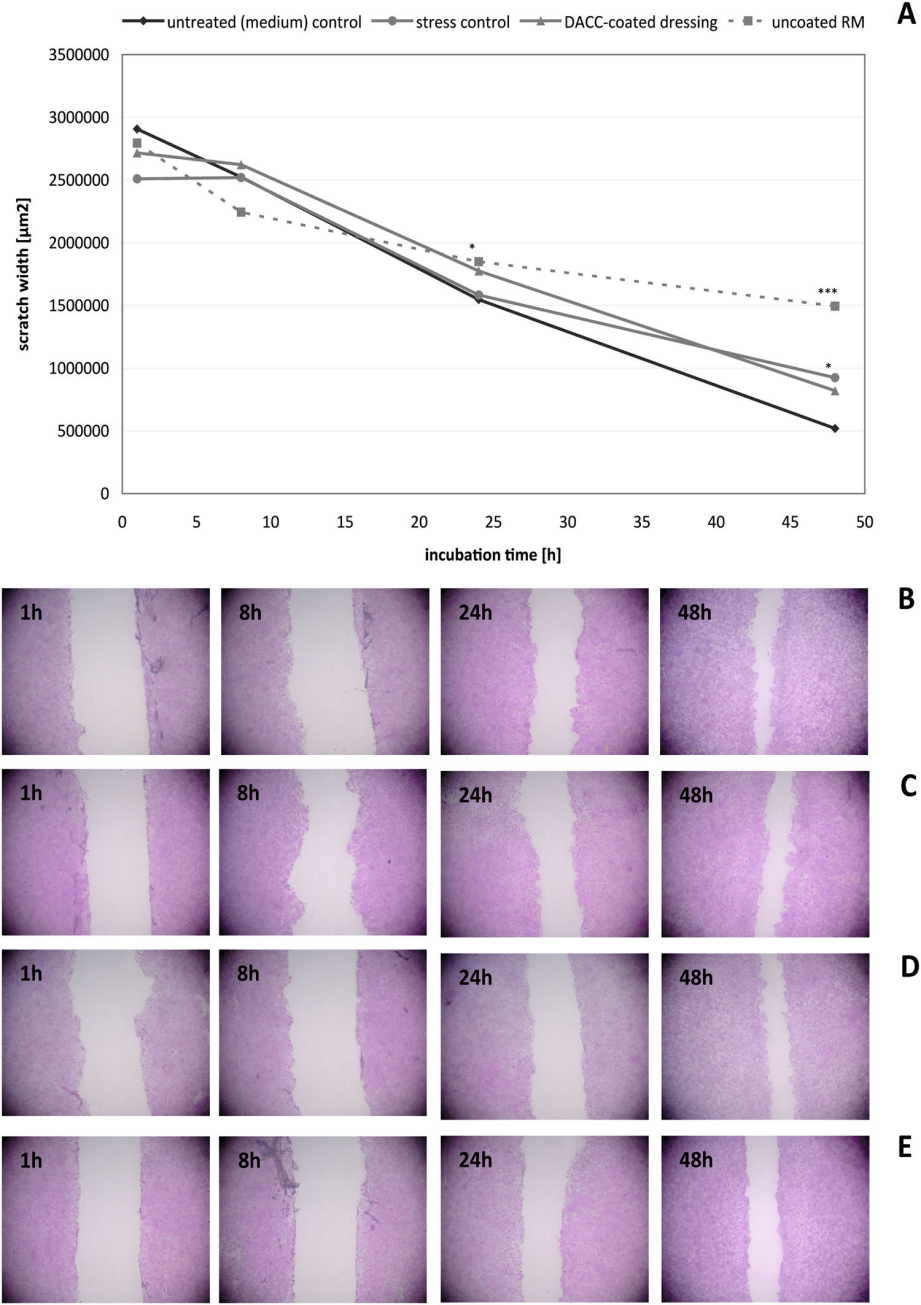
Progression of HaCaT scratch healing (**A**) in the untreated (medium) control (**B**) and the stress control (**C**) as well as under treatment with DACC (**D**) and untreated RM (**E**) after 1, 8, 24, and 48 hours (h). Asterisks indicate a significant deviation at the respective time point from the untreated control (*p < 0.05; **p < 0.01; ***p < 0.001)

**Fig. 4:**
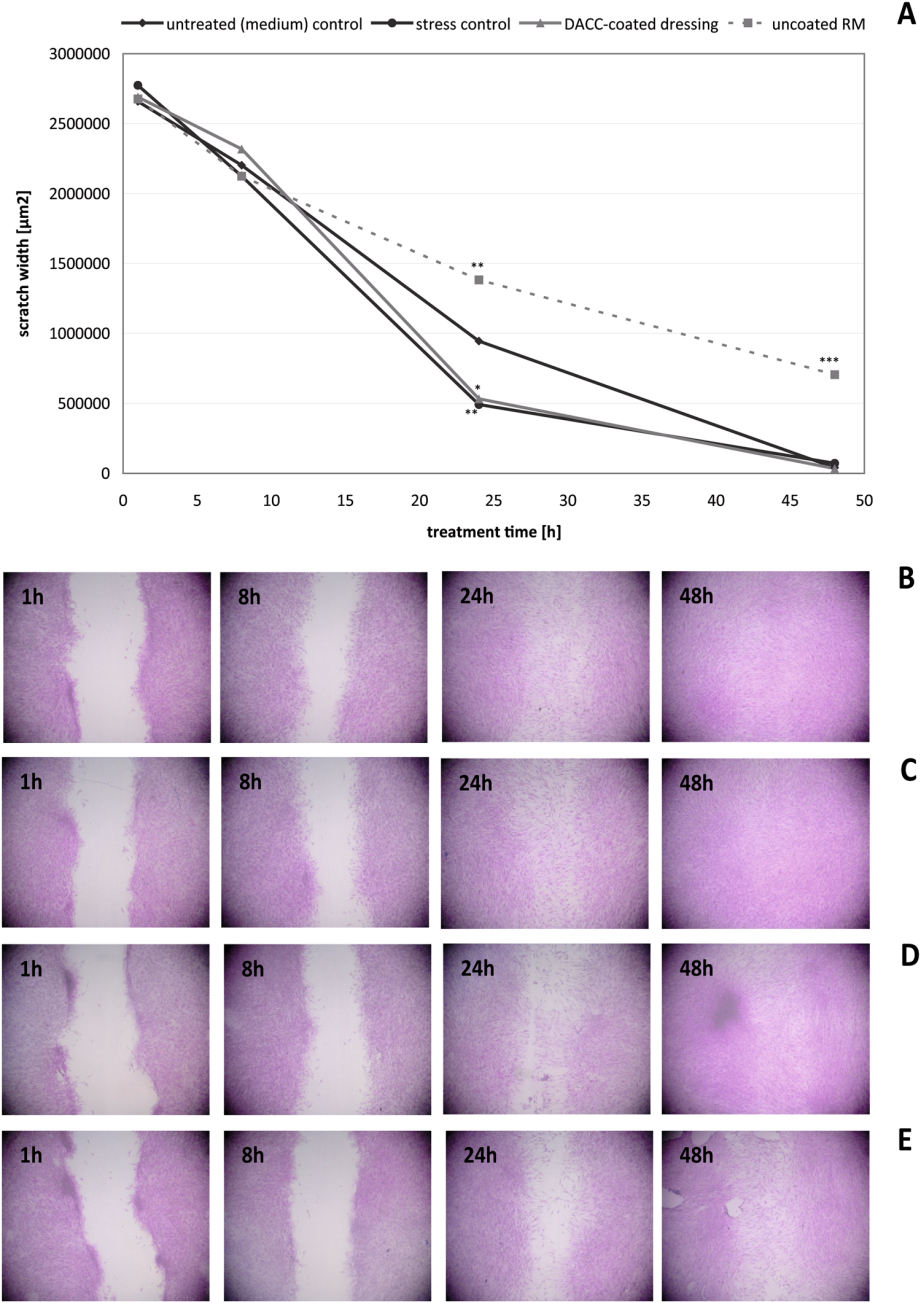
Progression of NHDF scratch healing (**A**) in the untreated (medium) control (**B**) and the stress control (**C**) as well as under treatment with DACC (**D**) and untreated RM (**E**) after 1, 8, 24, and 48 hours (h). Asterisks indicate a significant deviation at the respective time point from the untreated control (*p < 0.05; **p < 0.01; ***p < 0.001)

Mechanical scratch wounds of confluent HaCaT and NHDF cell layers served as models for studying potential effects of DACC-coated dressings on epithelial and dermal wound healing progression. It was found that DACC does not affect HaCaT scratch healing in vitro. The untreated control exhibited 80% scratch closure over 48 h (Fig. 3A, B). Under the stress control, scratch closure of approximately 60 % was observed (Fig. 3A, C), which was significantly lower than that of the untreated control (*p* < 0.05). During treatment with the DACC-coated dressing, the HaCaT keratinocytes showed normal cell proliferation and migration, resulting in 70% scratch closure over 48 h (Fig. 3A, D). This was slightly better than the stress control and not significantly different from the untreated control. In contrast, uncoated RM significantly reduced healing progression to only 47% (*p* < 0.001), and residual scratches at 48 h remained larger than those of the untreated control and those covered with DACC (Fig. 3A, E). Similar results were obtained for NHDF. Both, the untreated control and the stress control showed almost complete scratch healing over 48 h (Fig. 4A–C). DACC exhibited a positive healing progression with 99% scratch closure after 48 h (Fig. 4A, D) that was comparable to the untreated control and the stress control. DACC even showed a positive and increased healing progression at 24 h compared to the untreated control of 80% vs. 65%, respectively. No significant differences were observed for the DACC-coated dressing and stress control compared to the untreated control at 48 h. However, fibroblast scratch closure was significantly enhanced with these interventions compared to the untreated control at 24 h (*p* < 0.05). In contrast, uncoated RM was associated with decreased healing progression already at 24 h (*p* < 0.01) and resulted in only ~73% scratch wound healing at 48 h (*p* < 0.001) with scratches remaining open (Fig. 4A, E). Moreover, NHDF cell layer damage upon removal of uncoated RM was evident, which is in accordance with the observed cellular attachment to the fibers.

### Monitoring of wound healing progression in vitro by gene expression analysis

**Fig. 5:**
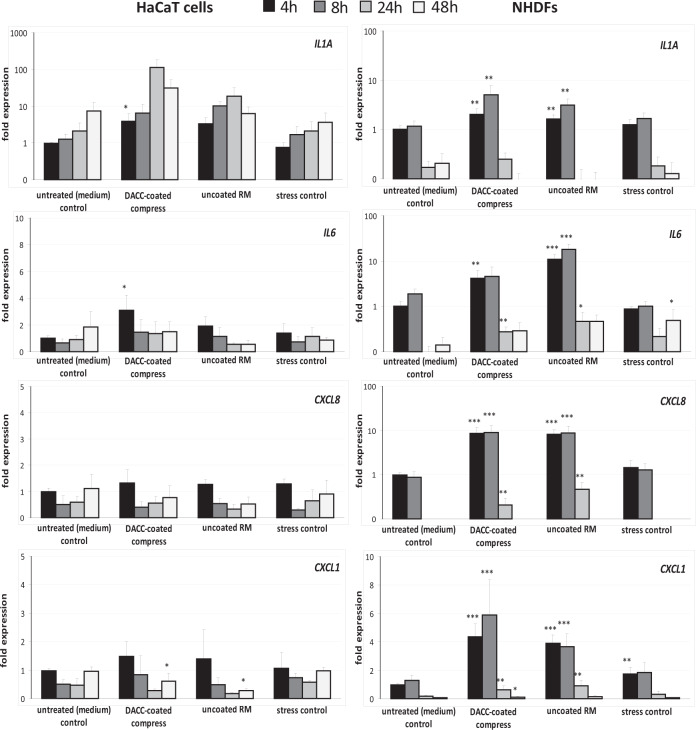
Treatment impact of DACC and uncoated RM on gene expression in keratinocytes (HaCaT cells) and fibroblasts (NHDF) during scratch wound healing compared to the untreated (medium) control and the stress control. Quantitative analyses of gene expression for the pro-inflammatory cytokine genes *IL1A*, *IL6*, *CXCL8*, and *CXCL1* were performed at 4, 8, 24 and 48 hours (h). Relative gene expression was normalized to the housekeeping gene *ACTB*. Data are presented as—fold gene expression compared to the untreated (medium) control at the start of the experiment. Asterisks indicate a significant deviation at the respective time point from the untreated control (*p < 0.05; **p < 0.01; ***p < 0.001)

**Fig. 6:**
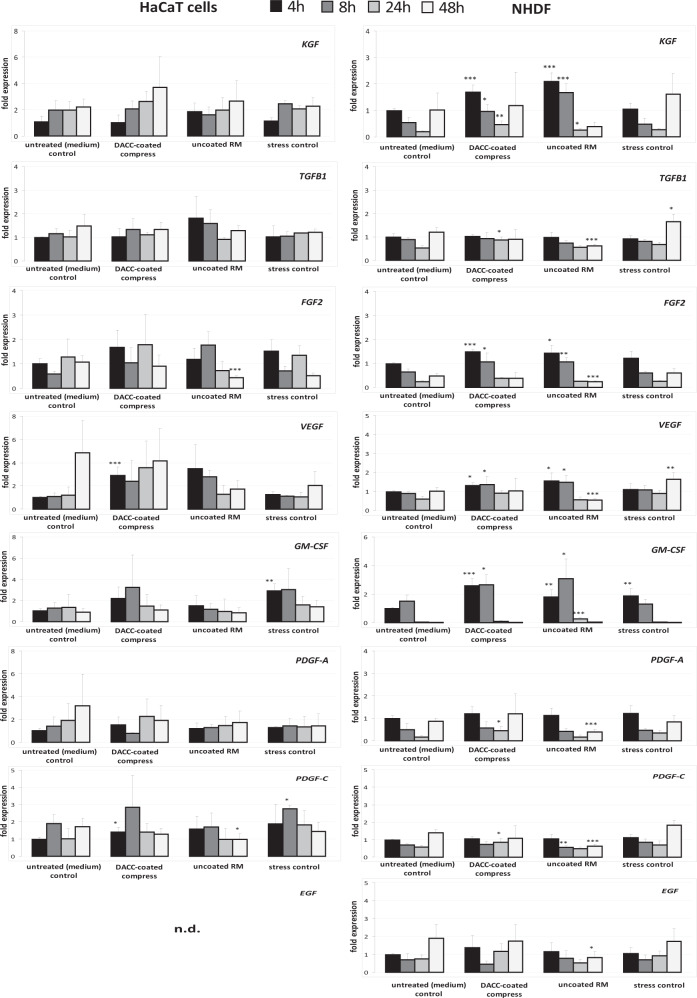
Influence of DACC and uncoated RM on gene expression in keratinocytes (HaCaT cells) and fibroblasts (NHDF) during scratch wound healing compared to the untreated (medium) control and the stress control. Quantitative analyses of gene expression for the growth factor genes *KGF*, *TGFB1*, *FGF2*, *VEGF*, *GM-CSF*, *PDGFA*, *PDGFC*, and *EGF* were performed at 4, 8, 24, and 48 hours (h). Relative gene expression was normalized to the housekeeping gene *ACTB*. Data are presented as—fold gene expression compared to the untreated (medium) control at the start of the experiment. n.d.—expression of *EGF* was not determined for HaCaT cells as keratinocytes do not produce this growth factor. Asterisks indicate a significant deviation at the respective time point from the untreated control (*p < 0.05; **p < 0.01; ***p < 0.001)

**Fig. 7:**
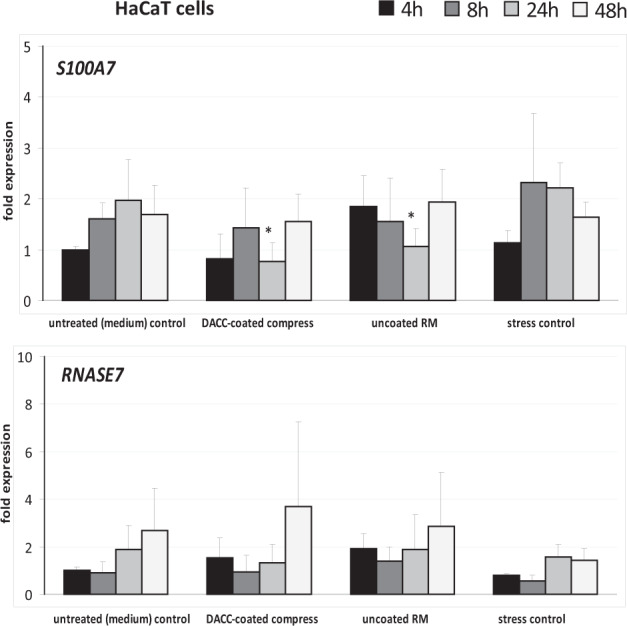
Gene expression of antimicrobial peptides under treatment with DACC and untreated RM during HaCaT scratch wound healing compared to the untreated (medium) control and the stress control. Quantitative analyses of gene expression for the antimicrobial peptide genes *S100A7* and *RNASE7* were performed at 4, 8, 24, and 48 hours (h). Relative gene expression was normalized to the housekeeping gene *ACTB*. Data are presented as—fold gene expression compared to the untreated (medium) control at the start of the experiment. Asterisks indicate a significant deviation at the respective time point from the untreated control (*p < 0.05; **p < 0.01; ***p < 0.001)

**Fig. 8:**
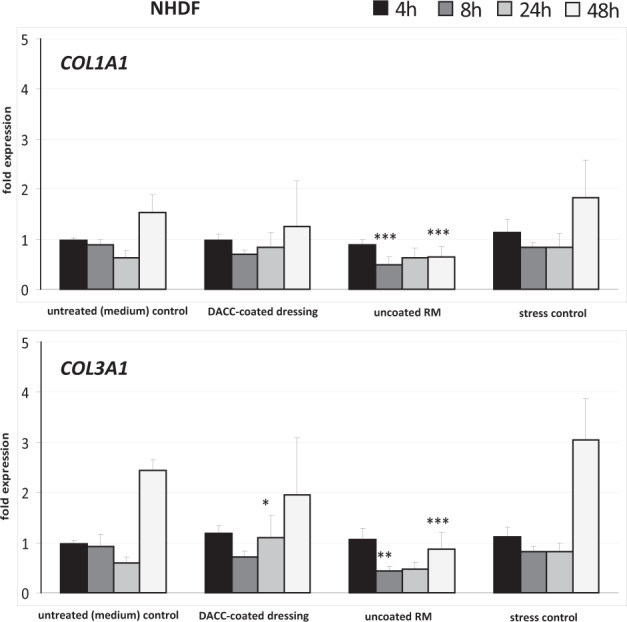
Collagen gene expression under treatment with DACC and uncoated RM during NHDF scratch wound healing compared to the untreated (medium) control and the stress control. Quantitative analyses of gene expression for collagen genes *COL1A1* and *COL3A1* were performed at 4, 8, 24, and 48 hours (h). Relative gene expression was normalized to the housekeeping gene *ACTB*. Data are presented as—fold gene expression compared to the untreated (medium) control at the start of the experiment. Asterisks indicate a significant deviation at the respective time point from the untreated control (*p < 0.05; **p < 0.01; ***p < 0.001)

In addition to observation of scratch wound closure, the wound healing process was monitored by gene expression analysis. Here, effects of DACC-coated and uncoated RM samples on cell responses as regards inflammation, induction of growth factor and collagen transcripts, as well as gene expression connected to antimicrobial defense were evaluated in vitro. Expression of *IL1A* by HaCaT cells under DACC and uncoated RM was significantly increased by a factor of approximately three-times compared to untreated control and stress control and peaked after 24 h with over 10-times the amount of transcripts (*p* < 0.05). In NHDF, the increase of *IL1A* transcripts was more moderate and limited to the first 8 h (Fig. 5). The increase of *IL6* in HaCaT cells was significant yet far more moderate compared to *IL1A* and limited to the first 4 h (*p* < 0.05). In contrast, transcript levels were significantly augmented in NHDF by approximately four-fold under DACC and over tenfold under the uncoated RM (*p* < 0.01). Similar observations were made for *CXCL8* and *CXCL1* gene expression under DACC and uncoated RM in NHDF, with levels particularly increased over 24 h (*p* < 0.05). In contrast, *CXCL8* levels were not affected in HaCaT cells and while *CXCL1* gene expression was found to be slightly elevated at 4 h, it was significantly decreased under DACC and uncoated RM at 48 h (*p* < 0.05) compared to the untreated control (Fig. 5). The expression of *KGF* and *VEGF* was induced over time in HaCaT cells by DACC and *FGF2* levels had almost doubled as early as 4 h (Fig. 6). However, these changes were statistically not significant compared to the untreated control. Levels of *PDGF-C* and *GM-CSF* transcripts were increased at early time points (*p* < 0.05). In NHDF there was an early and significant (*p* < 0.05) increase of *KGF*, *FGF2*, and *VEGF* gene expression (Fig. 6). Moreover, *GM-CSF* levels had almost doubled compared to the untreated control (*p* < 0.05). *TGFB1*, *PDGFA*, and *EGF* gene expression was hardly affected by incubation with the DACC-coated dressing (Fig. 6). *GM-CSF* levels seemed to be predominantly increased by mechanical stress and decreased to control levels after 24 h, although, the uncoated RM exhibited a prolonged induction of *GM-CSF* in NHDF (*p* < 0.001). In contrast, the expression of *FGF2* (*p* < 0.001), *VEGF* (*p* < 0.05), *PDGF-A*, and *PDGF-C* (*p* < 0.05) in HaCaT was decreased under treatment with uncoated RM compared to the untreated control at 48 h (Fig. 6). Similar observations were made for *KGF*, *TGFB1* (*p* < 0.001), *FGF2* (*p* < 0.001), *VEGF* (*p* < 0.001), *PDGF-A* (*p* < 0.001), *PDGF-C* (*p* < 0.001), and *EGF* (*p* < 0.05) after treatment of NHDF with uncoated RM at 24 to 48 h (Fig. 6). The DACC and uncoated RM samples did not affect the antimicrobial peptide gene expression by HaCaT cells of *RNASE7* (Fig. 7) nor that of *S100A7* except at 24 h, where DACC was associated with a slightly decreased expression of *S100A7* compared to the untreated control (*p* < 0.05). The gene expression of *COL1A1* and *COL3A1* by NHDF was not negatively affected by DACC compared to the untreated control (Fig. 8). Moreover, a *COL3A1* level twice as high as the untreated control was observed at 24 h (*p* < 0.05). In contrast, these two collagen genes were distinctly less expressed in uncoated RM treated samples at both 24 h (*p* < 0.01) and 48 h (*p* < 0.001).

## Discussion

In this study, the effect of a DACC-coated wound dressing on wound healing was assessed using an in vitro scratch wound assay with human keratinocytes (HaCaT cells) and NHDF. Fibroblasts together with keratinocytes ensure maintencance of the barrier function of normal skin. When the skin defense is broken, fibroblasts, and keratinocytes enter the wound area to reform skin tissue. The mechanical scratch wounding of confluent monolayers, often referred to as the scratch wound assay, serves as a model to study cell migration at the wound margins, as well as by de novo synthesis and deposition of newly formed matrix components [19]. Such models are particularly interesting because they mimic the effects of actual skin injury on cell proliferation and migration, as well as the inflammatory processes elicited by a mechanically induced scratch wound of cell layers. Recently, the model has been shown to be adaptable and thus applicable to the investigation of the effects of wound dressings on scratch closure as a model for healing wounds [21]. Cytotoxic effects of dressings applied to a wound may diminish healing rates by decreasing cell viability, cell proliferation and cell migration. The DACC-coated dressing was found to exhibit high cell compatibility in vitro, similar to both the untreated control and a stress control. The DACC coating on the knitted fabric from cellulose acetate makes the surface hydrophobic, i.e., it renders low surface energy and a contact angle with water of >110° [24]. The DACC-coated dressing was further compared to the basic, uncoated material (declared here as uncoated RM) with a hydrophilic fabric surface. Uncoated RM also had no effect on the metabolic activity of HaCaT cells and NHDF, although, cells were observed to attached rapidly to its uncoated fibers. This is in accordance with the observation that human cells adhere more easily to hydrophilic surfaces via attachment of proteins like vitronectin and fibronectin [25]. This result was verified by evidence of cellular ATP on the uncoated RM at levels significantly higher than those on the DACC-coated dressing (almost 25 times). Consequently, this resulted in observable damage to the cell monolayers in the scratch wound assay after RM removal. In contrast, ATP levels found on DACC-coated fibers were very low and there was no visual damage to the cell layers. Hence, it can be concluded that the hydrophobic DACC-coating of the basic material minimizes cell attachment and thereby increases safety as regards application to tissues. Moreover, such a hydrophobic surface functionalization is thought to convey antimicrobial properties by binding microorganisms through hydrophobic interactions [25]. So far, DACC-coated dressings have shown promising results in several clinical studies on the prevention and treatment of wound infections [6, 7, 26]. An effect of DACC on the complex biological process of wound healing is overall possible. Recently, Falk and Ivarsson reported improved proliferation and increased migration of fibroblasts treated with DACC in an experimental, cell culture model [22], which reflects the findings in this study despite experimental differences. Here, the primary aim was to determine the cellular safety of the DACC-coating in a wound healing situation so complete cell culture media were chosen to allow for optimal wound closure in the controls. During treatment with DACC-coated samples, keratinocytes demonstrated normal cell proliferation and migration, leading to a scratch closure of ~70% over 48 h comparable to the untreated control. Similar results were obtained for NHDF; DACC even showed a positive and increased healing progression at 24 h compared to the untreated control of 80% vs. 65%, respectively, and complete scratch closure (99%) after 48 h. In contrast, the uncoated RM significantly reduced wound healing progression over 48 h (*p* < 0.001) and open scratches persisted, probably due to cell attachment to the hydrophilic fiber surface. The DACC-coating did not affect the healing processes negatively and supported normal wound closure in vitro. The wound healing process was also monitored by gene expression studies using RT-PCR. Here, the effects of DACC-coated samples and uncoated RM on cell responses associated with inflammation, growth factor induction and collagen synthesis, as well as antimicrobial defense were evaluated in vitro. Controls showed increased expression of the pro-inflammatory cytokine gene *IL1A* over time in HaCaT keratinocytes, while its expression was found to decrease over time in fibroblasts. Similarly, *CXCL1* and *CXCL8* expression declined over the observation period in both cell types. *IL6* expression showed no significant changes in HaCaT keratinocytes yet was diminished in fibroblasts. Studies have shown an immediate elevation of inflammatory mediator gene expression of, such as *IL6*, *IL1A*, or *CXCL8*, after wounding in vivo [27–29] as well as in vitro [30], which induce reparative effects like growth factor release and cell migration towards the wound site. Keratinocytes can actively participate in the inflammatory response by releasing pro-inflammatory cytokines and chemokines [14, 15]. Fibroblasts adopt similar functions, producing IL-6 and IL-8 in response to danger signals in addition to their major role in ECM production [31]. In contrast to these findings, a decline in gene expression for most of the inflammatory cytokines was observed in this study. This can be explained by the prolonged observation period starting at 4 h and lasting until 48 h after wounding, when the general inflammatory reaction triggered by cell layer wounding can be expected to decline gradually and the reparative processes predominate. For instance, Hotta et al. demonstrated induction of *CXCL8* in keratinocytes up to 4 h after treatment and a subsequent decline in gene expression [32]. Downregulation of the inflammatory response generally has been associated with faster healing and less scar formation [33, 34]. Hence, it was of interest to investigate whether the improved healing response observed clinically with the DACC-coated dressing [6, 7, 26] might partly be due to effects on inflammatory processes of the cells involved in wound healing. However, gene expression of *IL1A*, *IL6*, *CXCL8*, and *CXCL1* by HaCaT cells and fibroblasts was found to be significantly increased by the DACC-coated dressing as well as the uncoated RM compared to the untreated control and also exceeded that of the stress control. Therefore, it is unlikely that positive effects on wound healing observed here stem from a decreased inflammatory response. The interchange between keratinocytes and fibroblasts is regulated in a paracrine manner via growth factors and plays a particularly vital role for completion of wound closure [35, 36]. Therefore, the hypothesis that improved induction of growth factors stimulates wound closure was also investigated here. No distinct changes over time were noted in *TGFB1* and *FGF2* levels in keratinocytes in response to the untreated or stress control. A slight increase in fold expression (2- to 4 times) was observed for *KGF, PDGFA, PDGFC* and *VEGF*. Mechanical stress further induced the early increase in *GM-CSF* transcript levels. Growth factor gene expression in fibroblasts differed to some extent from that of keratinocytes with no distinct changes observed for *TGFB1*, *VEGF*, and *PDGFC* over time while decreased levels of *KGF*, *FGF2*, *GM-CSF*, and *PDGFA* gene expression were noted. Interestingly, after 48 h a return to starting levels of gene expression at 4 h was found for *KGF* and *PDGFA*. An induction of *EGF* expression was also observed over time. Timely and spatially differential dispersion of growth factors plays a major role in the proliferative process during wound healing and is regulated in both, a cytokine-dependent and paracrine fashion [37]. For instance, IL-1 acts not only on keratinocytes, but also activates nearby fibroblasts and increases the secretion of KGF, which subsequently stimulates keratinocyte migration and proliferation [38]. Other cytokines, such as TNF-α induce secretion of the FGF family by fibroblasts [39], which supports fibroblast migration and deposition of ECM components [40]. Another important signaling molecule produced by both keratinocytes and fibroblasts is TGF-β, the expression of which is upregulated directly upon wounding [41]. However, TGF-β has also been implicated in the formation of fibrotic tissue, such that the reduced production of TGF-β may help to prevent fibrosis [42]. Here, we only observed a triggering of *TGFB1* expression in fibroblasts by the stress control at a late time point, which was accompanied by a slight increase of *COL1A1* and a significant rise in *COL3A1* expression corresponding to the reformation of the fibroblast layer in the scratch wound assay. Increased collagen expression has been implicated in the formation of keloid scars [43], but no changes compared to the untreated control were found for the dressings tested. Thus, it is unlikely that the DACC-coating used for functionalization of the wound dressing induces an unwanted fibrotic response during wound treatment. The DACC-coated dressing was found to slightly induce *KGF*, *VEGF*, and *FGF2* expression in HaCaT keratinocytes and fibroblasts while *TGFB1*, *PDGFA*, *PDGFC*, and *EGF* were less affected. Overall, growth factor gene expression profiles in both cell types, as well as collagen gene expression in fibroblasts, showed no negative effects of the DACC-coated dressing on the wound healing process emphasizing its high cell compatibility. Moreover, small positive effects on growth factor gene expression were observed that could explain the clinically observed supportive effects of DACC-coated dressings in wound treatment. In contrast, *COL1A1* and *COL3A1* expression was distinctly decreased in cells treated with the uncoated RM; this either explains the inhibited scratch closure or is due to the observed scratch progression inhibition. Moreover, significantly diminished levels of *FGF2*, *VEGF*, *PDGF-A*, and *PDGF-C* were observed in both cell types after 48 h of treatment with uncoated RM. The gene expression of *KGF*, *TGFB1*, and *EGF* was further reduced in NHDF treated with uncoated RM, which could account for the inhibited scratch healing progression. Finally, and importantly, gene expression of the antimicrobial peptides *S100A7* and *RNASE7* by HaCaT keratinocytes was monitored under the influence of the DACC-coated dressing and uncoated RM. Both genes are upregulated upon wounding, and the antimicrobial peptides are responsible for the protection against evading pathogens and have also been implicated directly in the wound healing process [44, 45]. In line with this, a distinct increase in their expression was observed over time in the scratches here. There were no apparent gene expression differences with physiological consequences after treatment with DACC-coated dressing samples compared to controls. This confirms that antimicrobial effects observed clinically with the DACC-coated wound dressing can be attributed solely to an inhibitory effect of DACC-coating on bacteria rather than the induction of the innate immune defense system in keratinocytes.

## Conclusion

Physical binding of microorganisms by wound dressings presents a novel concept on preventing wound infection, which may be crucial in the light of increasing global antimicrobial resistance [1]. Coating fibers with DACC renders them hydrophobic and conveys high binding capacity for bacteria [4]. At the same time, however, it is of utmost importance to avoid negative effects on the wound healing process. Here, for the first time, a comprehensive methodology for reviewing cell viability and proliferation, as well as modeling wound healing that includes gene expression analysis in vitro was used to study the effects of a DACC-coated dressings on the wound healing process. Clearly, such models are restricted to human cell cultures and cannot take into account the complex, multicellular, and three-dimensional interactions that occur during wound healing [21]. Moreover, the materials would initially interact with a host’s inflammatory cells in vivo and consequently trigger crucial signaling pathways in dermal fibroblasts. Generally, wound healing is a complex biological process not easily studied in vitro nor in vivo. Nonetheless, using the comprehensive in vitro methodology described here, it was possible for the first time to distinctly differentiate between the effects of DACC-coated dressings and uncoated RM, which might eventually wield a clinical influence. Furthermore, DACC-coating was found to be safe to use and may positively support wound healing by physical interaction.

## Data Availability

The data in this study are available on request from the corresponding author.
